# Effectiveness of inpatient versus outpatient complex treatment programs in depressive disorders: a quasi-experimental study under naturalistic conditions

**DOI:** 10.1186/s12888-019-2371-5

**Published:** 2019-12-02

**Authors:** Martin Driessen, Philipp Schulz, Silvia Jander, Hedda Ribbert, Stefanie Gerhards, Frank Neuner, Steffi Koch-Stoecker

**Affiliations:** 1 Klinik für Psychiatrie und Psychotherapie, Evangelisches Klinikum Bethel, Remterweg 69-71, 33617 Bielefeld, Germany; 2 Department of Psychology, Faculty of Psychology and Sports Science, Work Unit Clinical Psychology and Psychotherapy, Bielefeld University, 33615 Bielefeld, Germany

**Keywords:** Depression, Outpatient treatment, Inpatient treatment, Clinical trial, Effectiveness

## Abstract

**Background:**

Due to long waiting periods for outpatient psychotherapy and the high resource requirements of inpatient treatment, there is a need for alternative treatment programs for patients with depressive disorders. Thus, we investigated the effectiveness of the “Bielefeld Outpatient Intensive Treatment Program of Depression” (BID) in comparison with a typical inpatient treatment program by using a prospective quasi-experimental observational study. We assumed (i) that both complex programs are effective in pre-post analyses after 6 weeks and (ii) that inpatient treatment is more effective compared with the outpatient program.

**Methods:**

Four hundred patients with depressive psychopathology – a majority with depressive episodes (ICD-10 F3X) - took part in the BID and 193 in the inpatient program. Different self- (i.e., BDI) and expert measures (i.e., MADRS) of psychopathology at baseline (t1) and 6 weeks later (t2) were applied to examine treatment effects.

**Results:**

Treatment effects were high in separate analyses of both groups with Cohen’s *d* ranging from 1.10 to 1.76., while ANOVA comparative analyses did not reveal any significant differences between both treatment settings nor did a set of independent covariates analyzed here. Response rates of BDI (*p* = .002) and MADRS (*p* = .001) were higher in the outpatient group. Results indicate BID not to be inferior compared to an inpatient program, although diverging pathways to treatment, higher rates of clinical recurrent depressive disorders and severe episodes as well as lower rates of employment and partnership in the inpatient treatment group have to be considered.

**Conclusion:**

Outpatient intensive treatment programs may represent a solution for patients needing more than a treatment session once per week but less than a complex inpatient or day clinic program.

## Background

Depressive disorders represent a wide-spread mental disorder with a lifetime prevalence of 16–20% [1–4] and are considered to be one of the most striking public burdens of disease [5]. Chronic courses of depressive disorders are frequent as well as long times of incapacity to work [6, 7]. Thus, the access to effective guideline-oriented treatments should not only be generally available but also early after onset of depression [8]. However, long waiting periods (at least 3 to 6 months) especially for psychotherapy are widespread [9]. In addition, many health care systems focus on either low-intensity and low-cost outpatient treatments with most often not more than one short session per month or maximum once per week psychotherapy sessions and on the other hand on complex hospital (full or day clinic), highly expensive treatments.

However, there are alternatives to purely outpatient psychotherapeutic or inpatient forms of treatment for depressive disorders such as complex integrated outpatient care models [10]. These treatment models consist e.g. of cognitive-behavioral therapy sessions, if indicated psychopharmacologic treatment and social interventions by social workers, sport exercises, as well as regular monitoring of the treatment progress. Aims of such integrated care models are increasing quality and cost-efficiency of treatment [11]. A moderate number of such models were designed in the mental health field, but only few were realized for more than two to 3 years [12, 13], although Gunn and Diggens [14] in an overview reported that multi-professional approaches with structured treatment plans and planned post-intervention care do not only lead to increased inter-professional communication but may also have a positive impact on treatment outcomes in depression. Up to now and to the best of our knowledge, there is almost no evidence on the effectiveness of integrated care models in comparison to inpatient treatments (e.g. [15]), thus, more research in this field is needed.

In the city of Bielefeld, Germany, a city with about 340.000 inhabitants, an integrated care model contract was established in 2007 (valid up to now), in which the outpatient service of our psychiatric clinic, regional unions of physicians and psychotherapists working in private practices (“Medi-OWL “and “APP”) and a work group of health insurances, covering about 30% of individuals in the region (“ARGE BKK OWL”) participate. The aforementioned new established integrated care model is called „Bielefeld Intensive Outpatient Depression Program “(BID), which primarily focusses on the treatment of patients with depressive disorders. BID offers an outpatient, intensive, and multimodal treatment over 6 weeks, which is described below. On the other hand, the clinic offers a traditional 7 days per week inpatient complex treatment program for patients with depression without a fixed number of weeks but often also lasting about 6 weeks (see below for more details).

Considering previous investigations having shown that outpatient treatments were effective in (chronic) depressive disorder [16–19] we assumed that both programs are clinically effective in pre-post analyses after 6 weeks of treatment. However, given that the inpatient treatment offers substantially more specific and non-specific therapies in 7 days per weeks as well as permanent availability of specialist personnel and immediate help in the event of crises [20], we assumed that it is more effective compared with the outpatient program.

## Methods

### Design and procedure

This is a prospective quasi-experimental observational, i.e. non-randomized, controlled study [21], in which two samples of patients with depressive disorders are compared who are treated in an inpatient versus outpatient complex intervention program. Thus, it represents a 2 × 2 factorial design with two times of assessments (and 6 weeks of interventions in between) as well as considering independent demographic and clinical variables as covariates.

**Fig. 1:**
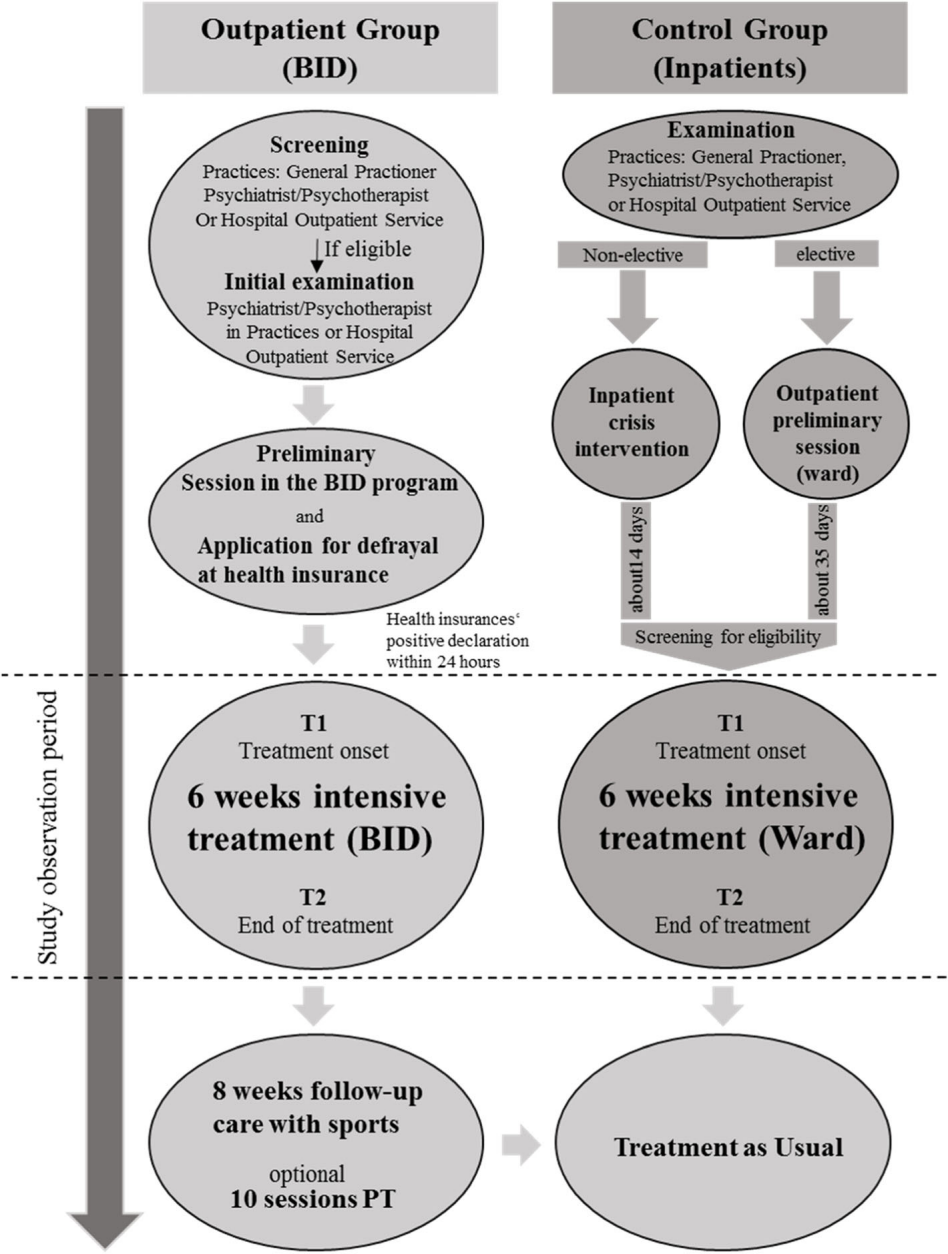
Elements of the Bielefeld Outpatient Intensive Depression Program (BID) and inpatient depression program as well as pathways into, within and out of the programs. Note: PT = Psychotherapy; T1/T2 = Times of measurement

Noteworthy, patients pathways into these two programs were somewhat different due to the fact, that the BID represents an integrated care program for patients with a specific health insurance (BKK; see above as well as Fig. 1): While in both programs patients could be transferred by private practices (general practitioners, psychiatrists, psychotherapists), consultation centers or the hospital’s outpatient services directly, participants of the inpatient program could be also transferred from a hospital crisis intervention unit when primarily admitted as a case of emergency.

Within the contract for participation in the BID program patients gave their written consent into data analysis for an evaluation study while the inpatient participants gave their written consent separately from the admission contract. Standards of good clinical practice and the demands of the Declaration von Helsinki were fulfilled.

### Inpatient complex treatment program for depression

The multimodal treatment program consists of weekly extensive visits (patient, psychiatrist, psychologist, nurse), pharmacotherapy if indicated, cognitive behavioral and in some cases additional psychodynamic psychotherapy in single and group settings, training of social competencies (once per week), sport exercises (twice per week), relaxation training (twice per week), imagination, occupational, art and/or music therapy (each therapy offered twice per week), as well as 20–30 min sessions with the primary nurses (at least once per week). Each patient receives his/her own schedule with individual frequencies and combinations of treatment services. However, each patient receives at least one (or two) psychotherapeutic single and one group session per week, one psychoeducation group session, one (to three) at least 30 min lasting session with the individual primary nurse, as well as one or two other treatment services (e.g. exercise therapy and imagination) per day.

### Bielefeld outpatient intensive treatment program BID

The BID as an integrated care program focusses on treatment of 18 to 65 years-old patients with a depressive episode with (recurrent) depressive or bipolar disorders (ICD-10 F31.x, F32.x, F33.x) as well as adjustment disorders (F43.21) and Dysthymia (F34.1) [22], if clinical severity of overall psychopathology is comparable. Major aims of treatment are reducing depressive symptoms, improving self-effectiveness, re-establishing the capacity to work (and return to job), and avoiding hospital admissions for mental health reasons. Figure 1 describes the pathway of the patients through the program.

In the inscription procedure, a screening is performed by the institution with the primary contact with the patient, and – if positive – a primary examination follows by a psychiatrist, who from now on overtakes the role of a case manager (CM) with weekly contacts during the intensive treatment episode. If the psychiatrist confirms the diagnosis, a first contact with a psychologist of the BID program takes place in the hospitals outpatient service in order to check cognitive and language capacities, expectations, aims, and motivation of the patient. Finally, if the assumption of costs is declared by the health insurance (within 24 h), the six-weeks outpatient intensive treatment episode starts.

It consists of cognitive-behavioral group (twice per week) and single (once per week) therapy sessions, one session per week with the CM psychiatrist (monitoring the progress, psychopharmacologic treatment if needed), contact with the social worker with a frequency dependent of needs (e.g. rehabilitation issues, contacts to the employee), sport exercises twice per week (Jogging/Walking, gymnastics), and Qi Gong once per week. The time schedule covers daily 1 to 3 sessions from Monday to Friday.

The CBT group therapy according to Hautzinger und Schaub [23, 24] consists of three parts, each lasting 2 weeks: 1. Psychoeducation and enhancing activities, 2. cognitive therapy, and 3. Training of social competencies. In the single therapy sessions, these contents are individualized and deepened. During the 6 weeks program at least two case conferences take place for each patient.

Sport exercises and Qi Gong are continued after the intensive treatment period for further 8 weeks, and up to ten psychotherapy single sessions are optionally available, but this period is not issue of the present report.

### Sample

In both settings, 18 to 65 years old patients were included into the study, who fulfilled clinically assessed diagnostic criteria as reported above. Further inclusion criteria were sufficient German language capacities and the explicit wish of the patients to take part in the treatment. Exclusion criteria were comorbid psychotic disorders, current substance use disorders, neurocognitive disorders, and eating disorders.

**Fig. 2:**
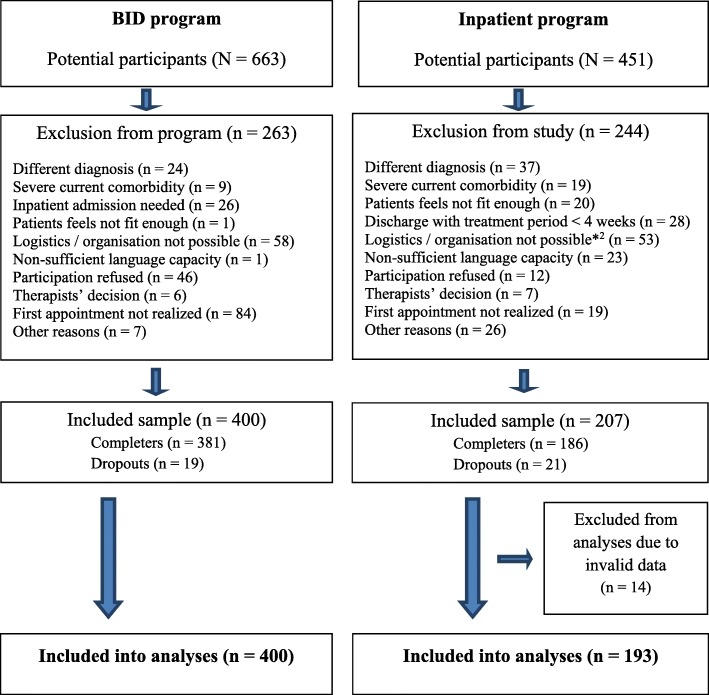
Flow-Chart reporting sampling in the two treatment groups

The sample size needed for detecting a small to medium effect in the two groups x two times design, i.e. Cohen’s *f* = .15, α error *p* = .05, a power of 95% was calculated as a total *n* = 148, i.e. *n* = 74 per treatment group (Program G*Power [25];. Figure 2 reports the development of the actual sample sizes in both treatment groups.

The samples at t1 consisted of 400 participants in the BID and 207 participants in the inpatient groups, from whom only 19 (4.8%) and 21 (10.1%), respectively, did not completed treatment.

### Assessments

At t1 diagnoses according to ICD-10 were clinically assessed by fully educated psychiatrists or clinical psychologists in both groups and, in addition, by using the German version of the International Checklists for ICD-10 [26]. Apart from assessment of demographic data at t1, the following instruments were used at t1 and t2 in both groups:

Depression: Beck Depression Inventory (BDI-II) [27, 28] and Montgomery Asberg Depression Rating Scale (MADRS) [29, 30].

Overall burden of psychopathology: Symptom Checklist SCL-90-R, German version [31], the Global Severity Index (GSI) is reported here.

General functioning: Global Assessment of Functioning Scale GAF (DSM-IV [32],).

### Analyses

For comparing completers and non-completers within both groups as well as for comparisons between groups χ^2^-tests and *T*-tests were performed. Treatment effectiveness within the two treatment groups were analyzed by using Cohen’s *d* basing on within-group pre-post T-tests. Effect sizes are interpreted according to the classification of Cohen [33] with *d* = 0.2 indicating a small, *d* = 0,5 indicating a moderate, and *d* > 0.8 indicating a large effect. In order to compare effectiveness between both treatment groups 2 (groups) × 2 (times) repeated measures ANOVAs were performed with group as between-subjects factor and time as within-subjects factor. Independent variables, which showed significant differences between groups at t1 were included as covariates. Analyses included interaction effects time x groups as well as time x group x covariates.

For the intention-to-treat analysis, we imputed missing data for outcome variables by using the expectation maximization procedure. The proportion of missing data on outcome variables ranged from 4.2% (BDI baseline) to 19.6% (GAF at t2). Littles’s missing completely at random-test revealed that missing outcome data were completely at random, χ^2^(211) = 228.60, *p* = .19. All analyses were repeated using listwise deletion method, yielding comparable results (data available on request). All analyses were performed by using the Statistical Software for the Social Sciences (SPSS Version 25).

## Results

### Sample characteristics

Completers and non-completers within the inpatient (*n* = 21 non-completers) and the BID group (*n* = 19 non-completers) did not differ significantly regarding age, gender, partnership, school education, current employment status, psychotropic medication, rate of recurrent depressive disorders and baseline scores in most outcome measures, i.e. all *p* > .09 (data available on request). In the BID group, non-completers were somewhat more often lower educated (χ^2^ = 4.52; df = 1; *p* = .03). In the inpatient group, non-completers had somewhat more often a mild to moderate depression (χ^2^ = 5.92; df = 1; *p* = .02) and showed a lower MADRS score at baseline (*t*(191) = 3.00, *p* = .003).

Table 1 reports demographic and clinical characteristics of the two treatment groups at t1. There were not any significant differences regarding age, gender, education, number of clinical psychiatric diagnoses and current suicidal thoughts. However, patients in the inpatient treatment group lived less frequently with a partner (χ^2^(1) = 18.55, *p* < .001), were less frequently employed (χ^2^(1) = 26.98, *p* < .001) suffered more often from recurrent depressive disorders (χ^2^(1) = 44.77, *p* < .001) and more often from current severe depressive episodes than patients in the BID group (χ^2^(1) = 170.86, *p* < .001). In addition, inpatients had a lower global functioning (GAF: *t*(591) = 11.51, *p* < .001) and took more often prescribed psychotropic medication, most often antidepressants (χ^2^(1) = 23.05, *p* < .001) than BID participants. However, groups did not differ in terms of self-reported or therapist-evaluated severity of depression (BDI: *t*(328.60) = − 0.54, *p* = .59; MADRS: *t*(591) = −.61, *p* = .57, for *M* and *SD* see Table 2).

**Table 1:** Sample characteristics at t1

		BID (*N* = 400)	Inpatient (*N* = 193)	
	Missings (n)	M (SD) or n (%)	M (SD) or n (%)	Statistics (df)
Female (%)	0	245 (61.3)	116 (60.1)	χ^2^ (1) = 0.07; *p* = .79
Referral, n (%)	19 (3.2)			
General Practitioner (private practice)		9 (2.3)	8 (4.2)	
Psychiatrist (private practice)		269 (70.2)	106 (55.5)	
Psychotherapist (private practice)		19 (5.0)	9 (4.7)	χ^2^ (6) = 94.11; *p* < .001
Outpatient Service (hospital)		65 (17.0)	22 (11.5)	
Hospital transfer		0	39 (20.4%)	
Counselling of Health Insurance		13 (3.4)	0	
Others		8 (2.1)	7 (3.7)	
Age; *M* (*SD*)	0	43.1 (11.2)	41.5 (12.2)	*t* (351.82) = 1.56; *p* = .11
Partnership; n (%)	21 (3.5)	240 (63.2)	85 (44.3)	χ^2^ (1) = 18.55; *p* < .001
Highest school education^a^; n (%)	14 (2.4)	173 (44.7)	86 (44.8)	χ^2^ (1) = 0.000; *p* = .98
Current employment; n (%)	21 (3.5)	277 (72.9)	98 (51.0)	χ^2^ (1) = 26.98; *p* < .001
Clinical diagnoses; n (%)	12 (2.0)			
Low depressive episode/Dysthymia/Adjustment disorder		62 (15.7)	3 (1.6%)	
Moderate depressive episode		291 (73.5)	61 (33.0)	χ^2^ (2) = 194.44; *p* < .001
Severe depressive episode		43 (10.9)	121 (65.4)	
Number of diagnoses; M (SD)		1.2 (0.4)	1.3 (0.5)	*t* (323.84) = − 0.97; *p* = .30
Severity of depression (low to moderate versus severe); n (%)	2 (0.3)	346 (86.5)	64 (33.5)	χ^2^ (1) = 170.86; *p* < .001
Recurrent depressive disorder; n (%)	1 (0.2)	67 (16.8)	81 (42.2)	χ^2^ (1) = 44.77; *p* < .001
Suicidal thoughts^b^; n (%)	45 (7.6)			
- Score 1		159 (43.6%)	90 (49.2%)	χ^2^ (3) = 6.41;
- Scores 2 and 3		19 (5.2)	17 (9.3)	
Psychotropic Medication^c^; n (%)	45 (7.6)	291 (80.6)	179 (95.7)	χ^2^ (1) = 23.05; *p* < .001

Legend: ^a^in Germany Abitur; ^b^Suicidal thoughts = Item 9 of the Beck Depression Inventory BDI-II (1 = “I have thoughts of killing myself, but I would not carry them out”, 2 = “I would like to kill myself”, 3 = “I would kill myself if I had the chance”); ^c^prescribed medication

**Table 2:** Pre-post measures and within-group effect sizes in the two treatment settings; *n* = 400 (BID) *n* = 193 (inpatients)

	Group	Time 1 *M* (*SD*)	Time 2 *M* (*SD*)	T-tests	Cohen’s *d*^1^
Severity of self-rated Depression (BDI)	BID	29.8 (9.5)	17.4 (10.5)	*t* (399) = 25.7; *p* < .001	−1.36
	Inpatient	30.3 (11.2)	20.2 (12.0)	*t* (192) = 18.4; *p* < .001	−1.36
Severity of depression assessed by the therapist (MADRS)	BID	26.6 (6.4)	13.9 (7.5)	*t* (399) = 32.5; *p* < .001	−1.76
	Inpatient	27.0 (6.3)	15.5 (4.7)	*t* (192) = 23.2; *p* < .001	− 1.49
Global Functioning (GAF)	BID	52.5 (8.6)	64.8 (11.5)	*t* (399) = −21.1; *p* < .001	1.25
	Inpatient	43.9 (8.3)	58.8 (7.7)	*t* (192) = −20.9; *p* < .001	1.45
Global burden of psychopathology (SCL-90-R GSI)	BID	73.3 (8.0)	62.7 (12.0)	*t* (399) = 20.2; *p* < .001	−1.33
	Inpatient	73.4 (8.4)	66.2 (11.1)	*t* (192) = 12.5; *p* < .001	− 1.10

Legend: ^1^*d* ≥ 0.2 small effect, *d* ≥ 0.5 moderate effect, d ≥ 0.8 large effect (marked)

In order to analyze potential heterogeneity within the inpatient group, we subdivided this sample in those patients who were admitted directly from home and those who were transferred from the crisis intervention unit: At t1 neither self-reported depression differed (BDI: direct admission *M* = 30.2, *SD* = 10.9; transfer *M* = 30.4, *SD* = 12.7; *t*(191) = − 0.08, *p* = .93) nor did therapist-rated depression (MADRS: direct admission subgroup *M* = 26.7 *SD* = 5.7; transferred subgroup *M* = 28.2 *SD* = 8.4; *t*(44.08) = − 1.06, *p* = .30). In addition, in the transferred subgroup the number of hospital days before transfer to the inpatient program was not significantly associated with the severity of depression at t1 (BDI: *r* = −.21, *p* = .23; MADRS: *r* = −.22, *p* = .19).

### Effectiveness of treatment

After 6 weeks of treatment pre-post analyses yielded in significant clinical improvements within both groups with strong effect sizes regarding all characteristics measured (i.e. Cohen’s *d* > 1) (Table 2).

Mixed repeated measures ANOVAs did not reveal any significant time x group interaction effects for the clinical measurements (see Table 3). In addition, the covariates severity of diagnoses, recurrent disorder versus single episode, medication (yes/no), family, and employment status did not have any significant influence on these results. In other words, we did not find any differences of effectiveness between the two treatment groups.

**Table 3:** Results of repeated measures ANOVAs and analyses of covariance comparing the treatment groups with independent covariates analyzed separately; sample sizes for main ANOVAs without covariates were *n* = 400 (BID), *n* = 193 (inpatients); df = 1 in dependent variables; df = 2 in covariates; error df = 515

Outcome	Effects of time	Effects of group	Interaction time x group	Interactions time x group x covariate	
Severity of self-rated depression (BDI score)	F = 23.01 *p* < .001	F = 0.21 *p* = .65	F = 1.33 *p* = .25	Severity^a^	F = 1.91; *p* = .15
				Recurrent^b^	F = 1.51; *p* = .22
				Family Status^c^	F = 1.16; *p* = .32
				Employment^d^	F = 1.93; p = .15
				Medication^e^	F = 1.56; *p* = .21
Severity of depression assessed by the therapist (MADRS)	F = 26.52 *p* < .001	F = 1.19 *p* = .28	F = 0.51 *p* = .35	Severity^a^	F = 0.51; *p* = .60
				Recurrent^b^	F = 0.72; *p* = .49
				Family Status^c^	F = 0.29; *p* = .75
				Employment^d^	F = 0.63; *p* = .53
				Medication^e^	F = 2.00; *p* = .14
Global Functioning (GAF)	F = 22.84 *p* < .001	F = 2.19 *p* = .14	F = 1.08 *p* = .30	Severity^a^	F = 1.29; p = .28
				Recurrent^b^	F = 1.01; *p* = .36
				Family Status^c^	F = 0.44; p = .65
				Employment^d^	F = 1.81; *p* = .17
				Medication^e^	F = 1.27; p = .28
Global burden of psychopathology (SCL-90-R GSI)	F = 26.72 *p* < .001	F = 0.00 *p* = .998	F = 0.10 *p* = .75	Severity^a^	F = 1.56; *p* = .21
				Recurrent^b^	F = 1.22; p = .30
				Family Status^c^	F = 1.06; p = .35
				Employment^d^	F = 2.46; *p* = .09
				Medication^e^	F = 0.64; p = .53

Legend:^a^Regarding diagnosis (low to moderate versus severe depressive episode); ^b^Second or more versus first episode; ^c^partnership yes/no; ^d^current employment yes/no; ^e^Psychotropic medication yes/no

Response rates (proportion of participants with a T2 score < 50% of that at T1) were also analysed focusing on the core depression characteristics. BDI self-rating data yielded in 41.8% responders in the outpatient and 28.8% in the inpatient group, indicating a larger rate of responders in the outpatient group (χ^2^ = 9.25, df = 1, *p* = .002). Interviewer-based MADRS scores also yielded a larger rate of responders in the outpatient group (46.6%) than in the inpatient one (30.9%) (χ^2^ = 13.06, df = 1, *p* < .001).

### Costs

While the inpatient treatment’s costs for 6 weeks were about 42 days x roughly 250 € = about 10.500 €, the BID program’s costs for the 6 weeks intensive treatment period (plus weekly excercises for further 8 weeks) were about 3000 € according to the contract with the health insurances.

## Discussion

The large treatment need of patients with depression is not sufficiently met in many countries. Especially, intensive and complex outpatient offers are missing for patients with moderate to severe disorders, for whom long periods of waiting and/or low intensity treatment modalities are not acceptable and/or not sufficient. Consequently, these patients are often admitted to mental hospitals and/or day clinics, although such high intensive treatments are not required in all cases. In addition, studies are missing, which evaluate and compare complex outpatient and inpatient programs for patients with depression. Here we present a complex inpatient program and the outpatient intensive program BID, both of them following the current national as well as international guidelines for the treatment of depression.

In a quasi-experimental design, we compared outcomes after 6 weeks and as assumed found that both programs are comparably high effective in within-groups pre-post analyses regarding the degree of the depressive syndromes, overall burden of psychopathology as well as global functioning. We had not expected the observed consistently high effect sizes with d > 1 [34], which may be due to the intensity of the programs. However, our data do not support our assumption that the inpatient program is more effective than the outpatient program BID, although the first one being more complex, intensive, and more expensive.

Noteworthy, inpatients differed from the BID patients at t1 in terms of current partnership, current employment, diagnoses of recurrent depressive disorders as well as current severe episodes (but not acute suicidality), and of the rate of subjects with psychotropic medication. It may be that recurrent episodes lead to social withdrawal or even generally to a lower global functioning [35]. On the other hand, the BID program may be more attractive for patients who are currently employed. However, all these characteristics were not found to significantly influence the results of repeated-measures covariance analyses. Noteworthy, we observed rather a low dropout rate during the 6 weeks of intensive treatment, which demonstrates its suitability for patients with depressive disorders.

As national and most international guidelines indicate, the efficacy and effectiveness of cognitive-behavioral therapy in depressive disorders as well as of combinations with antidepressive medication in severe depression were reported in a substantial amount of studies and meta-analyses [17–19, 36–38]. Group therapies were offered in both settings of the present study and were also shown to be effective [39] as well as did sport exercises and relaxation trainings [40, 41].

There are some examples for the effectiveness of integrated care programs in (mental) health problems [42–45]. However, to the best of our knowledge, there are not any investigations on outcome differences between outpatient and inpatient complex treatment programs [8]. Because of the inpatient treatment of depression covering 24 h of care and its greater complexity with more individualized services for the patients, we assumed its higher effectiveness compared to the less complex outpatient program. Our results do not support this assumption, but this conclusion may only be valid with some limitations: In the inpatient group, clinical diagnoses complemented by the International Diagnostic Checklists indicated a higher rate of current severe episodes as well as of recurrent depressive disorders than in the BID group. However, BDI and MADRS did not reveal substantial between-groups differences at t1. An advantage of BID is its higher flexibility with rather short waiting periods prior to treatment of no more than two to 3 weeks, so that the symptoms may be less often chronic and the prognosis more favorable [46, 47]. In the inpatient group, waiting periods varied substantially up to 8 weeks periods between the first contact and admission in most patients, while others were admitted to the crisis intervention unit and transferred to the treatment unit directly. This latter group of patients apparently needed at least an initial inpatient treatment because of a threatening crisis.

Regarding the differences of costs for the two treatment programs, cost-effectiveness relation of the outpatient BID program was substantially more favorable than that of the inpatient program.

### Limitations

There are some limitations with this study. First, only members of one group of general health insurances (the so called ARGE BKK OWL) had access to the BID program, so we cannot completely exclude an inherent systematic bias. Second, randomization to the treatment conditions was not possible. A randomized controlled trial is needed to exclude bias and support our results more definitely. Third, our data do not give information about ongoing effects beyond the end of the 6 weeks of treatment. Fourth, we are not able to analyze, which elements of the treatment programs are more or less important regarding the overall treatment effects. We can only state that the inpatient program included more therapeutic offers per week than the BID program. Fifth, we had no wait-list control group and consequently are not able to evaluate the relative superiority of treatments used here compared with placebo effects. However, the effect sizes determined in our study substantially exceeded effect sizes of wait-list control groups that were published in a meta-analytical review [48]. Finally, our results do not refer to patients with currently severe comorbid disorders because they were excluded from analyses.

## Conclusions

Even if considering a variety of limitations with this study as well as considering that a substantial proportion of patients with depressive disorders do primarily need a hospital admission, our results underline that an intensive and complex outpatient treatment over 6 weeks is not generally less effective than an inpatient program in a large proportion of patients concerned. In addition, costs for BID are substantially lower.

## Data Availability

The datasets used and/or analysed during the current study are available from the corresponding author on reasonable request.

## Abbreviations

ANOVA: Analysis of Variance
APP: Arbeitskreis niedergelassener Psychologischer Psychotherapeuten in Bielefeld (Workgroup of psychological psychotherapists in private practices in Bielefeld)
ARGE BKK OWL: Arbeitsgemeinschaft der Betriebskrankenkassen Ostwestfalen-Lippe (Work Group of health insurances Eastern Westfalia-Lippe)
BDI: Beck Depression Inventory
BID: Bielefeld Outpatient Intensive Treatment Program of Depression
CBT: Cognitive Behavioral Therapy
CM: Case Manager
GAF: Global Assessment of Functioning
MADRS: Montgomery Åsberg Depression Rating Scale
Medi-OWL: Verband der niedergelassenen Ärzte und Psychotherapeuten in Ostwestfalen-Lippe (Union of physicians and psychotherapists in private practices in Eastern Westfalia-Lippe)
SCL-90-R GSI: Symptom Checklist 90 items revised version; Global Severity Index
